# Investigations of potential microbial methanogenic and carbon monoxide utilization pathways in ultra-basic reducing springs associated with present-day continental serpentinization: the Tablelands, NL, CAN

**DOI:** 10.3389/fmicb.2014.00613

**Published:** 2014-11-13

**Authors:** Penny L. Morrill, William J. Brazelton, Lukas Kohl, Amanda Rietze, Sarah M. Miles, Heidi Kavanagh, Matthew O. Schrenk, Susan E. Ziegler, Susan Q. Lang

**Affiliations:** ^1^Department of Earth Sciences, Memorial UniversitySt. John's, NL, Canada; ^2^Department of Biology, University of UtahSalt Lake City, UT, USA; ^3^Department of Geological Sciences, Michigan State UniversityEast Lansing, MI, USA; ^4^Department of Earth Sciences, ETH-ZürichZürich, Switzerland

**Keywords:** serpentinization, Tablelands, carbon monoxide, methanogenisis, phospholipid fatty acids

## Abstract

Ultra-basic reducing springs at continental sites of serpentinization act as portals into the biogeochemistry of a subsurface environment with H_2_ and CH_4_ present. Very little, however, is known about the carbon substrate utilization, energy sources, and metabolic pathways of the microorganisms that live in this ultra-basic environment. The potential for microbial methanogenesis with bicarbonate, formate, acetate, and propionate precursors and carbon monoxide (CO) utilization pathways were tested in laboratory experiments by adding substrates to water and sediment from the Tablelands, NL, CAD, a site of present-day continental serpentinization. Microbial methanogenesis was not observed after bicarbonate, formate, acetate, or propionate addition. CO was consumed in the live experiments but not in the killed controls and the residual CO in the live experiments became enriched in ^13^C. The average isotopic enrichment factor resulting from this microbial utilization of CO was estimated to be 11.2 ± 0.2‰. Phospholipid fatty acid concentrations and δ^13^C values suggest limited incorporation of carbon from CO into microbial lipids. This indicates that in our experiments, CO was used primarily as an energy source, but not for biomass growth. Environmental DNA sequencing of spring fluids collected at the same time as the addition experiments yielded a large proportion of *Hydrogenophaga*-related sequences, which is consistent with previous metagenomic data indicating the potential for these taxa to utilize CO.

## Introduction

The geochemical composition of spring fluids can provide insight into serpentinization driven subsurface processes, but these indicators are often overlaid by microbial biogeochemical transformations, which remain poorly understood in these extreme ecosystems. Geochemically distinct fluids associated with serpentinization result from subsurface water-rock reactions whereby groundwater or ocean water reacts with ultramafic rock (peridotite), producing serpentine minerals, H_2_ gas, and hydroxide ions (Barnes et al., 1967; Sleep et al., 2004, 2011). The resulting fluids create an ultra-basic and reducing subsurface habitat. Present-day serpentinization occurs in slowly spreading marine ridges such as Lost City (Kelley et al., 2005), or at continental sites such as the Zambales ophiolite, Philippines (Abrajano et al., 1990); Semail ophiolite, Oman (Neal and Stanger, 1983; Fritz et al., 1992); Tekirova ophiolites, Turkey (Hosgormez, 2007); Cabeço de Vide Aquifer (CVA), Portugal (Marques et al., 2008); The Cedars, United States (Barnes et al., 1967); Del Puerto Ophiolite, California Coast Range, United States (Blank et al., 2009) and the Tablelands, Canada (Szponar et al., 2013). The continental sites share similar geochemical characteristics such as high pH, low E_h_, scarce electron acceptors, and limited dissolved inorganic carbon for autotrophic growth, which is considered extreme for most microbial life as we know it. However, geochemical measurements (Morrill et al., 2013) and genomics (Blank et al., 2009; Brazelton et al., 2012; Suzuki et al., 2013; Tiago and Verissimo, 2013) have demonstrated that life may exist in these environments. Additionally, Suzuki et al. (2014) have cultured organisms at high pH that correspond to highly abundant organisms in these serpentinizing environments, which are also the same taxa (Serpentinomas/Hydrogenophaga) observed at the Tablelands (Suzuki et al., 2014).

Dissolved or bubbling methane gas is common in fluids associated with sites of present-day serpentinization. The geologic setting and the water-rock reactions that occur at sites of present-day serpentinization in continental ophiolites create conditions amenable for the production of microbial, thermogenic, and/or abiogenic methane (Szponar et al., 2013). Geochemical indicators at most sites of serpentinization suggest that the methane produced is primarily non-microbial in origin (Neal and Stanger, 1983; Abrajano et al., 1990; Fritz et al., 1992; Hosgormez, 2007; Proskurowski et al., 2008; Szponar et al., 2013). However, there is still a potential for microbial production of methane at other sites of serpentinization. Methanogenic archaea have been noted at marine sites of serpentinization (Brazelton et al., 2006; Perner et al., 2007; Brazelton and Baross, 2010). At continental sites of serpentinization, methanogens were detected in the Del Puerto Ophiolite in the California Coast Range (Blank et al., 2009) and The Cedars (Suzuki et al., 2013), but the abundances, distributions, and diversity of methanogens have not been explored in these systems. In addition to genomic data, geochemical data from The Cedars did not preclude microbial methane production (Morrill et al., 2013). In previous studies at the Tablelands, sequences related to methanogens were extremely rare in the metagenomic dataset, and no genes indicative of methanogenesis were identified (Brazelton et al., 2012).

Microbial methane can be formed autotrophically (i.e., carbonate reduction, Equation 1), or heterotrophically (e.g., with organic acid substrates, Equation 2a and 2b).

(1) $${\text{CO}}_{2}+4{\text{H}}_{2}\to {\text{CH}}_{4}+2{\text{H}}_{2}O$$

(2a) $$3{\text{H}}_{2}+{\text{HCOO}}^{-}+{\text{H}}^{+}\to {\text{CH}}_{4}+2{\text{H}}_{2}\text{O}$$

(2b) $${\text{CH}}_{3}{\text{COO}}^{-}+{\text{H}}_{2}\text{O}\to {\text{CH}}_{4}+{\text{CO}}_{3}^{2-}+{\text{H}}^{+}$$

Organic acids, i.e., acetate and formate, are enriched at the Lost City hydrothermal field, a serpentinizing system (Lang et al., 2010). These organic acids may be present at sites of continental serpentinization and may be the carbon source for heterotrophic methanogenesis. CO_2_ for microbial methanogenesis via carbonate reduction (Equation 1) is potentially limited at high pH values because at pH values greater than 11, the dominant species of dissolved CO_2_ is not the biologically available bicarbonate (HCO^−^_3_), but instead the carbonate ion (CO^2−^_3_) that precipitates with the ample Ca^2+^ in serpentinizing systems. A recent study by Suzuki et al. (2014) found that *Serpentinomonas*, an organism adapted to the high pH and high Ca^2+^ concentrations characteristic of sites of serpentinization, used calcium carbonate for carbon fixation. These results suggest there maybe unknown pathways for CO_2_ reduction in high pH systems such as sites of serpentinization.

Microorganisms commonly found at sites of serpentinization may be able to use CO as the electron donor and carbon source. This pathway is catalyzed by the enzyme carbon monoxide dehydrogenase (CODH) (King and Weber, 2007), and gene sequences encoding for CODH were present in high abundance in the metagenome of the WHC2b spring (Brazelton et al., 2012). The CODH enzyme (A) is used to convert the available CO from the environment to CO_2_ (Equation 3), which is then used in further metabolic processes.

(3) $$\text{CO}+{\text{H}}_{2}\text{O}+\text{A}\to {\text{CO}}_{2}+{\text{AH}}_{2}$$

There is evidence of microorganisms at the Tablelands site with phylogentic affinity to the microorganism *Hydrogenophaga pseudoflava*, an autotrophic member of the Comamonadaceae family within the Betaproteobacteria (Brazelton et al., 2012). These microorganisms are facultative anaerobes that can grow autotrophically on H_2_ or fix CO when organic carbon is unavailable (Willems et al., 1989). *Hydrogenophaga*-related organisms and other organisms of the Comamonadaceae family have been detected at many sites of serpentinization where ultra-basic fluid mixes with either shallow groundwater or overland flow in pools of water (Schrenk et al., 2013; Suzuki et al., 2014).

An environment with high redox gradients exists at the surface where ultra-basic reducing springs associated with subsurface serpentinization discharge into pools of fluids that are open to inputs from shallow groundwater, overland flow, precipitation and the atmosphere. The microbial composition of these pools may be different from those of the subsurface. These mixing pools act as portals between the surface and the subsurface. The ultra-basic, reducing pool in the Tablelands, Gros Morne National Park, NL, CAN, is one location where carbon substrate utilization can be studied in one of the ultra-basic reducing portals between the serpentinizing subsurface and the oxic surface environments where mixing has been constrained (Brazelton et al., 2013; Szponar et al., 2013).

The Tablelands, also known as the Table Mountain massif, is one of four Ordovician ophiolites that make up the Bay of Islands Complex (BOIC) which runs along the west coast of Newfoundland, Canada in the Humber Arm Allochthon tectonic zone (Suhr and Cawood, 1993). The Humber Arm Allochthon is a mixture of deep sea sediments, mafic crustal material and mantle peridotites from the ancient seafloor that was assembled and obducted onto the eastern edge of the North American Craton about 500 Ma ago as the Iapetus Ocean was closing during the Taconian orogeny (Suhr, 1992; Suhr and Cawood, 1993). The mantle peridotite located at the Tablelands is classified as harzburgite and lherzolite, which have varying proportions of the ultramafic minerals olivine, orthopyroxene and clinopyroxene (Suhr, 1992). Recent serpentinization of the ultramafic body, which was most likely rejuvenated after the last glaciation (~12,000 years ago) when isostatic rebound created new cracks and fissures that exposed unreacted ultramafic rocks to groundwater, produced highly reducing (~ −600 mV) and ultrabasic (pH >11) waters rich in hydrogen gas, methane and other low molecular weight hydrocarbons (C_2_–C_6_) (Szponar et al., 2013). These fluids have been identified discharging from multiple active springs in pools of water (e.g., WHC1 and WHC2) surrounded by travertine deposits at the Tablelands providing evidence of present-day serpentinization.

The first objective of this study was to characterize the geochemistry and metabolic diversity of microorganisms within the mixed WHC2 pool and the ultra-basic water that discharges into the pool. The second objective was to determine the potential for microbial methanogenesis using inorganic carbon and organic acid substrates, and for microbial CO utilization using stable isotope probing and isotopic fractionation with non-labeled substrates. The results from this study provide empirical evidence in support of microbial CO utilization in agreement with earlier metagenomic studies. However, the results from this study do not provide evidence for microbial methanogenesis using the organic acids detected at sites of serpentinization or inorganic carbon within serpentinizing settings.

## Methods

### Site description and sampling procedure

**Figure 1:**
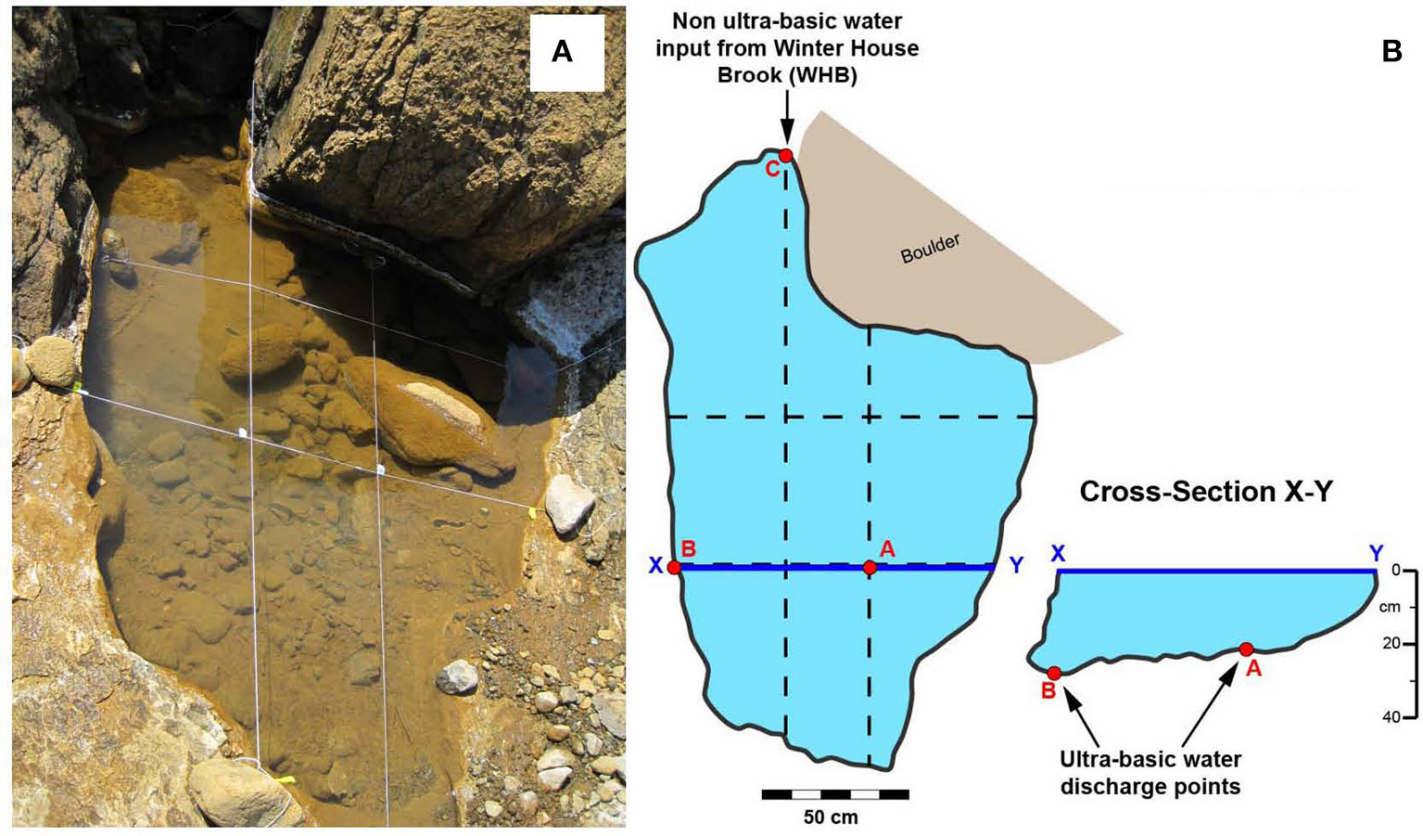
**(A)** Photograph of WHC2 ultra-basic pool. **(B)** Schematic planar and cross sectional sketches depicting the various water inputs into the WHC2 pool—A and B are locations of ultra-basic reducing water discharging into the bottom of the pool, C is the location of overland flow that trickles into WHC2.

Water and sediments were sampled from pool WHC2 (N 49°27 ′58.7 ″ W 057°57 ′29.2 ″). This pool of water was approximately 130 cm wide and 40 cm deep surrounded by travertine deposits and was exposed to the atmosphere at the surface. Within this pool two springs located at the bottom of the pool have been identified by low E_h_ values and are labeled A and B (i.e., WHC2A and WHC2B) (Figure 1). A site labeled C (WHC2C) represented a mixing site where overland water from a tributary of the brook was flowing into the highly reducing pool of water. Previous work by Szponar et al. (2013) and Brazelton et al. (2012) describe the various kinds of contamination at this site and demonstrated geochemically that the sources of fluid at the bottom of the WHC2 pool are distinct from any surface sources. In June, 2011, water and sediments were sampled from WHC2A for the bicarbonate and organic acid addition experiments. In October, 2012, an artificial dam was created diverting the overland flow from WHC2C, and the WHC2 pool was emptied. Ultra-basic reducing groundwater springs recharged the pool. The pool was emptied again multiple times before pool sediments and recharging waters at WHC2A were sampled for the CO addition experiments. Geochemical field parameters (pH, E_h_, sulfate, nitrate, phosphate, total inorganic carbon (TIC), δ^13^C_TIC_, dissolved organic carbon (DOC), δ^13^C_DOC_, CH_4_, δ^13^C_CH4_) were sampled for in 2011 and 2012.

Samples for total inorganic carbon (TIC) and dissolved organic carbon (DOC) were collected for both concentration and stable carbon isotope (δ^13^C) values in pre-combusted 40 mL amber vials spiked with mercuric chloride (HgCl_2_) and phosphoric acid (H_3_PO_4_) respectively. Samples for DOC were filtered through a 0.7 μm pre-combusted glass microfiber filter.

Dissolved CH_4_, H_2_, and CO were sampled using a modified syringe gas phase equilibration technique by McAuliffe (1971) and Rudd et al. (1974). Twenty milliliters of fluid was withdrawn with a 60 mL sterile syringe and shaken vigorously for 5 min with an equal volume of helium (He). This allowed for partitioning of the dissolved gas in the sample water into to the gas phase. The entire gas phase of 2 syringes (40 mL) was injected into a 30 mL serum vial, prefilled with degassed water and sealed with blue butyl stoppers. The dissolved gases in He displaced the water in the serum vial. Samples were fixed with 5 μL-saturated solution of HgCl_2_ to ensure there was no microbial growth in bottles. Dissolved CH_4_ was sampled for δ^13^C analysis by collecting 50 mL of fluid using a 60 mL sterile syringe and injecting samples into a pre-evacuated 125 mL serum vial fixed with HgCl_2_ and sealed with blue butyl stoppers.

Fluid was filtered through a sterile 0.22 μm (25 mm ID) MCE membrane filter and collected in clean 15 mL test tubes with a sterile 60 mL syringe for the analysis of sodium, potassium, sulfate, nitrate, and phosphate. Samples were kept frozen and dark until analysis. Thirty mls of water were collected in 50 ml falcon tubes for formate, acetate concentrations. These samples were kept frozen and dark until analysis.

Formate and acetate concentrations were measured at the Swiss Federal Institute of Technology (ETH) in Zurich, Switzerland on a Surveyor high performance liquid chromatograph (HPLC) with a P1000 pump and a PDA Plus 5 Diode Array Detector with a 50 mm cell path following the method of Albert and Martens (1997) with minor modifications. Adipic acid (20 nmol) was used as an internal standard and was injected into the sample before derivitization. Following derivitization an aliquot of sample (0.4 mL) was injected into a 1.5 cm Prevail Organic Acid C18 guard column (4.6 × 250 mm ID, 5 μm film thickness, Grace Davison Discovery Sciences) using an autosampler equipped with a 1 mL syringe. The mobile phase in the column was varying gradients of two solutions. Solvent A was 2.5% n-butanol, 50 mM sodium acetate, 2 mM tetrabutylammonium hydroxide, 50 mM tetradecyltrimethylammonium bromide with phosphoric acid which was used to adjust the pH to 4.5. Solvent B was pure methanol. The gradient program was as follows: 75:25 (A:B) for 23 min, then transitioned to a 50:50 mixture over 5 min, held at 50:50 for 5 min, then returned to a 75:25 mixture over 5 min and equilibrated at 75:25 for 5 min. Peaks were detected at 400 nm. The analytical error of individual measurements for acetate and formate was ±0.012 and ± 0.06 mg C/L, respectively. The reproducibility of duplicate analysis ranged from 4 to 48% RSD.

### Environmental DNA sequencing

Sequences of bacterial 16S rRNA genes from WHC2 pool samples collected in 2010 and 2011 have been previously published (Brazelton et al., 2013). For this study, additional environmental 16S rRNA gene sequences were collected from the WHC2 pool before and after it was emptied several times and allowed to refill. Filtering of the fluids and DNA extractions of the filters were conducted as described previously (Brazelton et al., 2013) and briefly summarized here. Sterivex filters were stored on wet ice in the field, frozen in liquid nitrogen as soon as possible, transported on dry ice, and stored at −80°C. DNA extractions were performed by lysis via freeze/thaw cycles and lysozyme/Proteinase K treatment and purified with phenol-chloroform extractions, precipitation in ethanol, and further purification with QiaAmp (Qiagen, Hilden, Germany) columns according to the manufacturer's instructions for purification of genomic DNA. Purified DNA was submitted to the Josephine Bay Paul Center, Marine Biological Laboratory (MBL) at Woods Hole for amplicon sequencing of the bacterial 16S rRNA gene via an Illumina MiSeq platform. Amplification and sequencing protocols developed at the MBL and an updated version of the protocol is available in Nelson et al. (2014). Quality-filtering of the sequences was conducted via the VAMPS pipeline (Huse et al., 2014).

Previously published environmental 16S rRNA sequences derived from three WHC2 samples collected in 2010 and 2011 (Brazelton et al., 2013) were generated at the DOE Joint Genome Institute with a MiSeq sequencing platform according to well-established protocols (Caporaso et al., 2012). The previously published JGI sequences and new sequences reported here from the MBL were generated with similar but non-identical methodologies. Therefore, we also re-submitted two of the 2010–2011 samples for which sequences were previously published (WHC2B-2010 and WHC2C-2011) to the MBL for sequencing with the updated MiSeq protocol to provide a more direct comparison with the 2012 samples.

Environmental 16S rRNA sequences were also collected from sediments at the bottom of the pool that were sampled in 2010 and 2011. These sediments were collected from the same location but at a different time than the sediments collected for the 2012 experiments described below. Sediments were sampled by suction with a sterile 60 mL disposable syringe. Sediments were allowed to settle in the syringe for several minutes, and the overlying fluid was expelled. Additional sediment was then sampled by suction into the same syringe, and the settling and fluid expulsion was repeated until the syringe was approximately half-full of sediment. The differentiation between spring- and water column-derived microbes using this method at this site has been previous published by Brazelton et al. (2013). Sediment samples were contributed to the Earth Microbiome Project (EMP; http://www.earthmicrobiome.org), which handled the DNA extraction via the MoBio PowerSoil kit and amplicon sequencing via the Illumina MiSeq platform as described by Caporaso et al. (2012).

**Figure 2:**
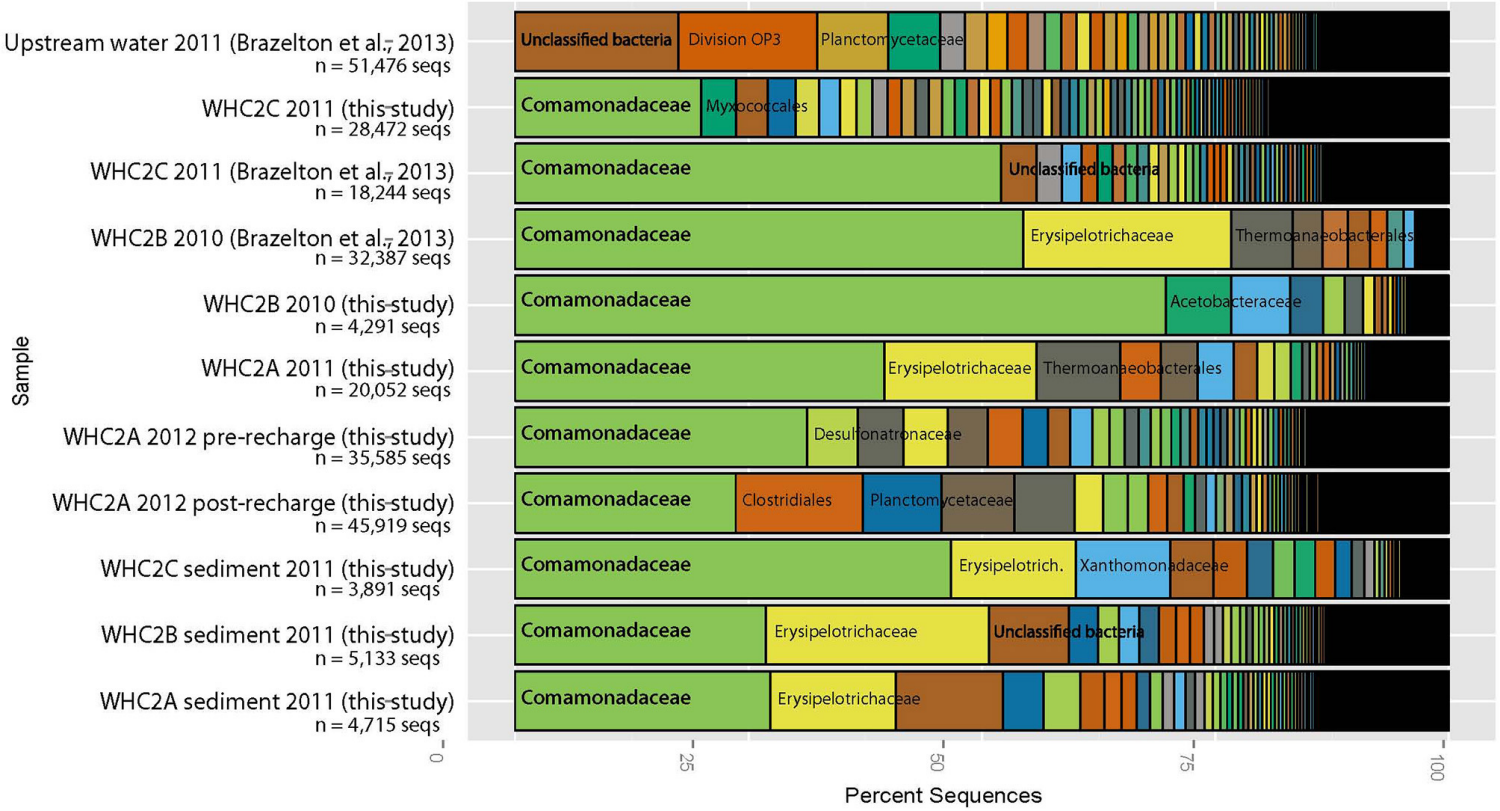
**Taxonomic classification of 16S rRNA amplicon sequences derived from fluids and sediments collected from the WHC2 pool and an upstream surface water source**. The Comamonadaceae family of Betaproteobacteria dominate both fluids and sediments from the WHC2 pool. Each colored slice represents the proportion of sequences assigned to each taxonomic family. The most abundant families are labeled; those that overlap multiple slices refer to the left-most slice where the family's name begins. For clarity's sake, not all families are labeled, but complete taxonomic classifications of all samples and sequences are available in the supplementary material.

Taxonomic classification of all sequences was performed using the SILVA reference alignment (SSURefv115) and taxonomy outline (Pruesse et al., 2007) using the mothur software platform (v.1.32.1) (Schloss et al., 2009). Figure 2 and supplementary material were generated in R (v.3.1.0) with the phyloseq package (v.1.9.4) (McMurdie and Holmes, 2013) using the taxonomic counts generated by mothur. Family-level classifications are shown in Figure 2 and supplementary material because the most common sequences could not be classified at lower taxonomic levels. MBL-generated sequences are freely available at the VAMPS database (http://vamps.mbl.edu) under the project code DCO_BRZ. All EMP-generated sequences are available via their database at http://www.microbio.me/emp under EMP Project ID 713: Serpentinite Seeps.

### Experimental sampling and approach

#### Methanogenesis experiments with organic acids and bicarbonate substrates

Sediment and water from WHC2A, the most ultra-basic reducing location in WHC2, were sampled anaerobically for the microbial carbonate reduction and organic acid fermentative methanogenesis studies (July, 2011). In the field, capped 12 ml exetainers (Labco Ltd., UK), which were prefilled with N_2_ gas, were lowered to the bottom of the WHC2 pool, the cap with a rubber septum was removed and ultra-basic reducing water displaced most of the N_2_ gas. The carbonate was of particular interest because, in a previous study, data indicated higher microbial concentrations within the carbonate sediment compared to the water itself (Szponar, 2012). While the exetainer was still under the water, ~2 ml carbonate sediment slurry was scooped into the exetainer. The exetainer was recapped underwater and stored upside down in the dark and transported to Memorial University. In the laboratory, 2 ml of water in each exetainer were displaced with an H_2_-rich headspace through the rubber septa. The exetainers were stored upside-down during the incubations.

To test for the potential of autotrophic or organic acid fermentative methanogenesis ^13^C labeled bicarbonate (autotrophic methanogenesis) or ^13^C labeled organic acid (fermentative methanogenesis) were added to the exetainers and the δ^13^C in CH_4_ and CO_2_ in the headspace was analyzed after incubation. These experiments were conducted with five labeled substrates, bicarbonate, formate, two isotopomers of position-specific labeled acetate (^13^CH_3_COO^−^ and CH^13^_3_COO^−^), and uniformly labeled propionate. For each incubation with a ^13^C labeled substrate, two controls were conducted, either by adding the non-labeled substrate (live non-labeled control), or by adding labeled substrate but inhibiting microbial activity by acidifying the fluid with 0.05 mL concentrated HCl to pH ~1 (killed ^13^C-labeled control). 0.3 mL of a 0.1 M solution of the unlabeled substrate were added to each exetainer. 0.1mL of a 0.001 M solution of the 99% ^13^C labeled substrate (Cambridge Isotope Laboratories, Cambridge, MA) were added to the live ^13^C-labeled and killed ^13^C-labeled treatments. After the substrate additions, the naturally occurring organic acids and inorganic carbon ranged from 1 to 4% of the total substrate concentration. After four months of incubation, stable carbon isotope values of CH_4_ and CO_2_ were measured.

#### CO utilization experiments

Water and sediment samples were collected at the Tablelands from WHC2A in October, 2012. Liquid and sediment samples were collected by completely submerging a 1L capped bottle under the water surface. At the bottom of the pool, the bottle cap was removed, and water and sediment were collected. In the laboratory, nine CO utilization experimental bottles were prepared (three live ^13^C-labeled substrates, three live non-labeled controls, and three killed non-labeled controls).

The CO utilization experiments were performed aerobically. Equal amounts of sediment (7 g) and ultra-basic water (70 mL) were added to each bottle. All nine bottles received 10 mL of mineral medium for chemolithotrophic growth (DSMZ medium 81), and were sealed with blue butyl septa and aluminum crimp seals. Contamination of volatile organic compounds present in the blue butyl stoppers was avoided by conditioning (i.e., boiling for 1 h in 0.1 N NaOH, followed by immersion in distilled water for 8 h, Oremland et al., 1987). All three killed controls received 1.4 mL of concentrated hydrochloric acid (HCl) which decreased the pH to ≤1. Two hundred microliters of non-labeled CO (i.e., CO with a natural abundance of ^13^C/^12^C, δ^13^C_CO_ = −44.4‰) were added to all bottles. Ten microliters ^13^C-labeled CO (99% ^13^C, Supelco) were added to the live ^13^C-labeled utilization experiments. After the CO substrate addition, the naturally occurring CO made up at most 0.02% of the total substrate concentration. Approximately 2 mL of lab air were added to all twelve bottles, to keep the experiments aerobic O_2_ and CO were distributed throughout the gas and liquid phases by continually shaking the bottles, having a 1:3 headspace to liquid ratio, and having a relatively large liquid surface area (12 cm^2^) in contact with the headspace. The concentrations of gases in the headspace were monitored over time on days 1, 2, 3, 8, 29, 50, and 59 days after CO was added. On day 59 the sediments and water were extracted for Phospholipid fatty acids (PLFA).

### Analytical methods

#### Gaseous concentrations and δ ^13^*C*

Concentrations of CO, CH_4_, and CO_2_ in the headspace of the experimental bottles were measured using a SRI gas chromatograph (GC) equipped with a flame ionization detector (FID) and a methanizer. Gases were separated using a Carboxen 1010 column (30 m × 0.32 mm, 30 μm) and the following temperature program: 40°C hold 6 min, ramp 25°C/min to 150°C, hold 6 min at 150°C. O_2_ concentrations in the headspace of the experimental bottles were determined using an Agilent 6890 GC equipped with a thermal conductivity detector (TCD) and using a N_2_ carrier gas. O_2_ was separated from other gases using the same column held at 40°C isothermal. A mixed gas standard (Scotty 14) was used for calibration curves. The Scotty Gas Standard contained 5.00% of O_2_, 5.00% of N_2_, 5.00% of CO, 4.01% of CH_4_, 4.00% of H_2_, and 5.00% of CO_2_ in a balance of He. The detection limits for CO, CH_4_, and CO_2_ in the gas phase were 10, 1.3, and 10 μmoles/L, respectively. Precision on triplicate injections of the standard was always less than 5% RSD.

The compound-specific carbon isotopic composition (δ^13^C) of CO, CO_2_, and CH_4_ was measured by gas chromatography-combustion-isotope ratio mass spectrometry (GC/IRMS; Agilent 6890N GC, Thermo Conflo III interface, Thermo Delta V+ IRMS). The gases were separated in the GC using a Carboxen 1010 capillary column (30 m × 0.32 mm). δ^13^C CO was analyzed using the following temperature program: 40°C hold for 6 min, then ramp 25°/min to 110°C, while CO_2_ and CH_4_ samples were analyzed using an isothermal temperature programme (110°C). Samples were taken from the microcosm bottles using a 0.025–0.5 mL gas syringe. Due to the change of concentrations, sample injection size varied (0.01–0.5 mL) per gas. Internal stable carbon isotope standards were used for CO, CH_4_, and CO_2_. The total analytical error associated with stable carbon isotope analysis by this method is ±0.5‰. Isotope ratios were reported in delta notation (δ^13^C) and were calculated relative to international standards V-PDB for carbon isotopes:

(4) $$\delta^{13}\text{C}=({\text{R}}_{\text{sam}}/{\text{R}}_{\text{std}}-1)$$

where R_sam_ and R_std_ are the ratio of heavy to light isotopes (i.e., ^13^C/^12^C) for the sample and standard, respectively (Coplen, 2011).

#### Phospholipid fatty acids extraction and identification

Phospholipid fatty acids (PLFA) were extracted according to a recently described protocol (Ziegler et al., 2013) based on the Bligh and Dyer process, modified for as follows: (1) experimental bottles were frozen and freeze-dried prior to extraction, and the extraction of polar lipids was conducted directly in the bottles; (2) the extraction was scaled down to a total extractant volume of 76 mL in 3 extraction steps; (3) samples were sonicated for 20 min prior to each extraction step, and decanted instead of centrifuged; and (4) phospholipids were eluated from silica columns with 6mL methanol (MeOH) and 6mL dichloromethane:MeOH:water (3:5:2) to optimize the recovery of phosphatidylcholines (Mills and Goldhaber, 2010).

Phospholipid fatty acids were transesterified into fatty acid methyl esters (FAMEs) by mild alkanolysis, spiked with an internal standard (2 μg ethyl eicosanoate), and quantified by gas chromatography/flame ionization detection (GC/FID; Agilent 6890A). FAMEs were separated on a BPX70 column (50 m length, × 0.22 mm inner diameter × 0.25 μm film thickness; SGE Analytical Sciences). The GC oven temperature was initially increased from 70 to 160°C at 10°C min^−1^, held for 5 min, then increased at 4°C min^−1^ to 260°C and held at 260°C for 15 min. FAMEs were identified with gas chromatography/mass specific detection (GC/MSD; Agilent 6890N GC with 5975C MSD), applying the same chromatographic conditions.

The compound-specific carbon isotopic composition (δ^13^C) of FAMEs was measured by gas chromatography-combustion-isotope ratio mass spectrometry (GC/IRMS; Agilent 6890N GC, Thermo Conflo III interface, Thermo Delta V+ IRMS). The same chromatographic conditions as used for GC/FID were applied except that a GC column with 0.32 mm internal diameter was used. Each sample was analyzed three times. IRMS performance was monitored by periodic analysis of an external reference standard containing 8 fatty acid methyl and ethyl esters (Mixture “F8.”A. Biogeochemical laboratories, University of Indiana) and constancy of the measured isotopic composition of the internal standard. The δ^13^C of the MeOH used for transesterification was determined with a TOC analyzer coupled to an isotope ratio mass spectrometer (OI Analytical Aurora 1030W; Finnigan Delta Plus XP; G. G. Hatch Stable Isotope Laboratory, University of Ottawa) and used to corrected the measured δ^13^C values of FAMEs for MeOH derived carbon.

### Statistical analysis

Differences in the abundance and composition of PLFA between live and killed treatments in the CO utilization experiments were tested using Welch's *t*-tests. Concentration or composition of PLFA were tested for significant differences between live bottles (both ^13^C-labeled and non-labeled) and killed bottles. The calculations were conducted using Libre Office V. 4.1.3.2. Due to the low number of replicates we report all *p* < 0.1.

## Results

### Geochemical characterization of WHC2

Aqueous geochemical parameters of the water at the WHC2A discharge point were determined in 2011 and 2012, and the recharge water (i.e., new pool water after repeated emptying of the pool) was also sampled at the WHC2A discharge location in 2012 (Table 1). The geochemical parameters (e.g., pH, dissolved H_2_, and dissolved CH_4_ concentrations and δ^13^C values) of the pool water changed very little from 2011 to 2012. Nutrient concentrations, nitrate and sulfate, and TIC concentrations in the recharge water were all very low compared to the adjacent Winter House Brook (Table 1). The recharge water had similar pH, dissolved H_2_, dissolved CH_4_, and phosphate values to the pool waters. The concentrations of TIC, nitrate, and sulfate, however, were lower in the recharge water compared to the pool water, while organic acid concentrations were higher in the recharge water compared to the pool water (Table 1).

**Table 1:** **Geochemical characterization of ultra-basic pool, spring recharge, and brook from 2011 to 2012**.

	**WHC2A**			**Winter house brook**	
	**2011^a^**	**2012**		**2011^a^**	**2012**
	**Pool**	**Pool**	**Recharge**		
pH	12.4	12.2	12.3	7.6 (±0.7)	8.1
E_h_ (mV)	−690	–	–	+415 (±26)	–
H_2_ (μmol/L)	585 (±25)	471 (±60)	476 (±50)	–	<d.l.
CO (μmol/L)	<d.l.	<d.l.	<d.l.	<d.l.	<d.l.
Sulfate (μmol/L)	8.54 (±0.10)	13.6	3.79	2.60 (±0.01)	11.6
Nitrate (μmol/L)	0.242 (±0.005)	2.29	1.24	3.06 (±0.05)	3.16
Phosphate (μmol/L)	0.842 (±0.632)	0.411	0.442	8.00 (±1.68)	0.474
Acetate (μmol/L)	–	25.4 (±1.2)	62.2 (±6.3)	–	2.10 (±0.63)
Formate (μmol/L)	–	1.11 (±0.44)	28.9 (±1.11)	–	<d.l.
TIC (μmol/L)	91.6 (±33.3)	233 (±175)	124 (±6)	671 (±142)	654 (±1)
δ^13^C TIC (‰)	−14.7 (±0.9)	−17.1 (±0.7)	−23.5 (±0.3)	−1.7 (±0.8)	−4.3 (±0.2)
DOC (μmol/L)	33.3 (±16.7)	–	–	38.3 (±16.7)	–
δ^13^C DOC (‰)	<d.l.	–	–	−27.1 (±0.6)	–
CH_4_ (μmol/L)	20.0 (±0.6)	26.2 (±1.2)	25.6 (±0.6)	–	<d.l.
δ^13^C CH_4_ (‰)	−27.1	−25.9	−27.7	–	–

a *With the exception of nutrient concentrations all other 2011 was reported in Szponar et al. (2013). –, data are not available; <d.l., represents analytes there were below the detection limits of the analysis*.

### Bacterial diversity of WHC2

Betaproteobacteria belonging to the Comamonadaceae family dominated the WHC2 pool water samples collected in 2012 (Figure 2), which is consistent with previously published results from WHC2 pool samples collected in 2010 and 2011 (Brazelton et al., 2013). Metagenomic sequences affiliated with this Comamonadaceae-affiliated taxon, for which (Suzuki et al., 2014) have proposed the genus name *Serpentinomas*, encode proteins involved in CO utilization and carbon fixation via Rubisco (Brazelton et al., 2012). Interestingly, the Comamonadaceae sequences comprised a somewhat smaller proportion of the bacterial community in the post-recharge pool (i.e., immediately after it was emptied several times), suggesting that they are not enriched in the subsurface (Figure 2). These sequences are very rare in the upstream surface water source, so they are not derived from surface water, either. These observations indicate that the Comamonadaceae organisms thrive in the mixing zone where subsurface ultra-basic fluids meet surface waters and perhaps atmospheric gases, which is consistent with previous results (Brazelton et al., 2012, 2013).

The bacterial compositions of carbonate-rich sediments collected from the bottom of the WHC2 pool also appear to represent a mixture of subsurface and surface materials. Each sediment sample has a large representation of Comamonadaceae, indicating that both sediments and overlying fluids are potential habitats for these organisms.

### Methanogenesis experiments using organic acids and bicarbonate substrates

**Figure 3:**
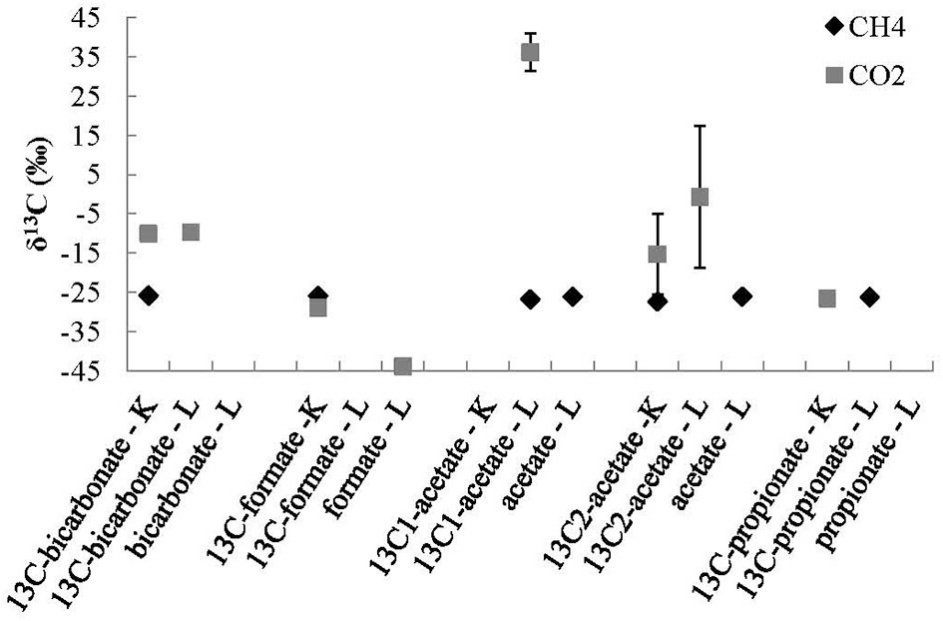
**Stable carbon isotope values of CH_4_ and CO_2_ products of ^13^C-labeled and non-labeled experiments where ^13^C-labeled bicarbonate, and organic acids were added to each treatment where K represents a killed control, L represents a live experiments, and 13C represents where ^13^C-labeled substrate was added**. The errors bars are the standard deviations of averages of duplicate experiments.

CH_4_ was detected in only 7 of the 15 experimental treatments (Figure 3). The CH_4_ was detected in live and killed treatments, as well as ^13^C-labeled and non-labeled substrate addition treatments. All CH_4_ had similar values, averaging −26.7 ± 0.7‰, including the ^13^C-labeled substrate addition experiments. The average δ^13^C_CH4_ value from the experiments was indistinguishable from the average δ^13^C_CH4_ value, −26.9 ± 0.9‰, (Table 1) determine from field samples. Therefore, there is no clear evidence of microbial production of CH_4_ using the substrates added in these treatments.

The concentration of CO_2_ was below the detection limit in many of the experiments, this was expected in the live treatments because the dominant species of inorganic carbon at high pH values is the carbonate ion, not the CO_2_ gas. CO_2_ was detected in all of the acidified killed control experiments, this was also expected because the dominant species of inorganic carbon at low pH values is CO_2_ gas (Figure 3). The CO_2_ measured in the ^13^C-labeled bicarbonate addition experiments, was more enriched in ^13^C than the TIC measured from the field samples (Table 1), reflecting the ^13^C-labeled bicarbonate that was added to each of these bottles.

In the killed ^13^C-labeled organic acid addition controls, the δ^13^C of the CO_2_ was similar δ^13^C of the TIC sampled from the recharge in the field (Table 1). In the live ^13^C-labeled acetate treatments, where the carboxyl-carbon (CH^13^_3_COO^−^) was labeled with ^13^C there was a production of CO_2_ in one replicate extetainer that was enriched in ^13^C (δ^13^C_CO2_ = +36.1‰) compared to other organic acid addition experiments (Figure 3). The very ^13^C enriched CO_2_ in one of the live ^13^C_C1_-labeled acetate addition treatments suggests that the carboxyl-carbon of the acetate molecule was converted to CO_2_ via heterotrophic respiration in that experimental bottle.

### CO utilization experiments

**Figure 4:**
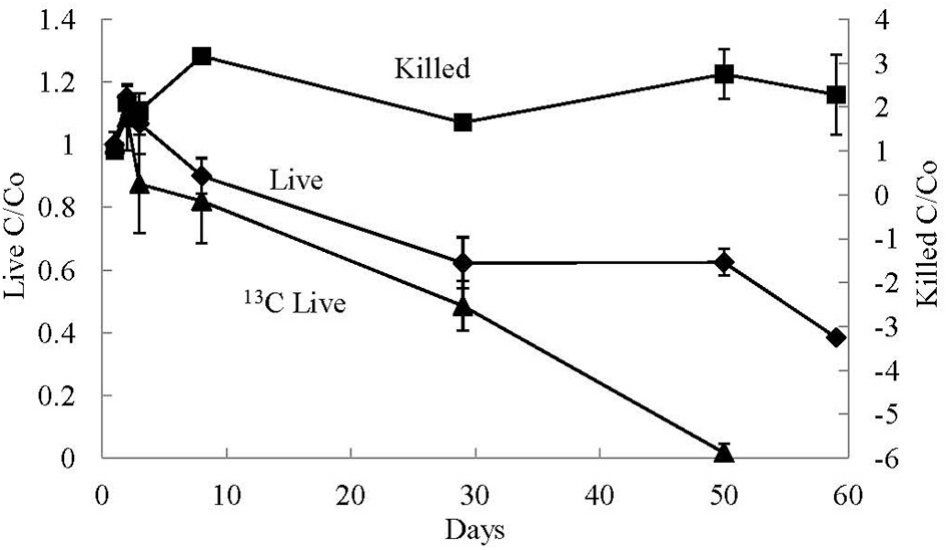
**Average relative gaseous CO concentrations (C/Co) measured over time in the CO addition experiments**. Experimental treatments included killed control (Killed), live non-labeled (Live), and ^13^C-labeled CO (13C Live). The error bars are the standard deviation of the average value of the triplicate treatments.

CO concentrations were monitored in all of the nine CO utilization experimental bottles (three live ^13^C-labeled substrates, three live, and three killed non-labeled controls). The headspace was sampled for CO concentrations on days 2, 3, 4, 9, 30, 51, and 60 of the experiment. Concentration changes relative to day 2 measurements are shown in Figure 4. The concentrations of CO slightly increased in all live experiments on day 3, probably due to incomplete CO equilibration on day 2. Subsequently, CO concentrations decreased in all of the live bottles. By day 60, there was no detectable CO in the live ^13^C-labeled experiments. In the live non-labeled experiments the concentration of CO decreased by 59% in one bottle, and 65% in another. The 3rd live non-labeled was accidentally vented on day 30, such that no data could be obtained after day 9 for this experiment. However, on day 9 the concentration of CO in this bottle had decreased by 15%, comparable to the other non-labeled bottles.

Similar to the concentrations of CO in the live experiments, the CO concentrations in the killed controls increased on day 3. However, unlike the live experiments, the CO concentrations in all of the killed bottles remained high for the remainder of the experiment. All of the increases and decreases were replicated in all three killed bottles. Overall the concentrations in the killed controls were always greater than the concentration measured on day 2 of the experiment.

O_2_ was present in the headspace of all CO addition experiments; however, the O_2_ concentrations in the headspace decreased over time in the live experiments, such that by day 60 there was on average 29 ± 10 μmoles/L of O_2_ gas in the headspace of the live CO addition experiments and 136 ± 27 μmoles/L of O_2_ gas in the headspace of the killed control experiments, such that there was approximately 79% less O_2_ in the live experiments compared to the killed controls on day 60. Therefore, the CO microbial utilization most likely occurred in an oxygenated environment.

**Figure 5:**
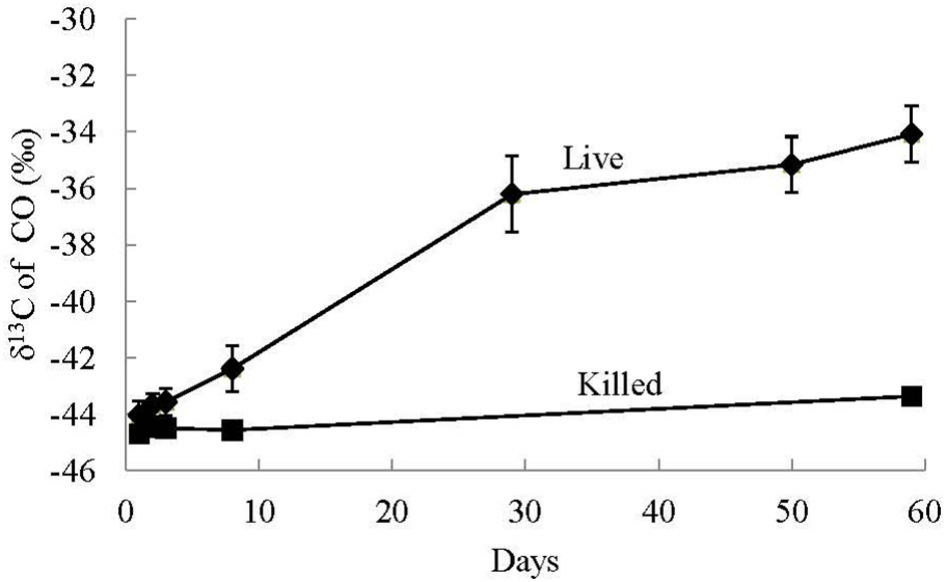
**Average stable carbon isotope values of CO in the headspace of non-labeled CO utilization experiments of live and killed control treatments**. The error bars are the standard deviation of the average value of the triplicate treatments. Significant isotopic enrichment of ^13^C in CO was only observed in the live experiments and not the killed controls.

The average starting δ^13^C of CO in the non-labeled experiments was −44.4 ±0.5‰. Over the course of the experiments the δ^13^C of CO in the live experiments became less negative with an average increase of 10 ± 0.3‰ between day 2 and day 60 (Figure 5). This isotopic enrichment in ^13^C corresponds to a 59–65% decrease in the CO concentrations in these experiments. Conversely, over the course of the killed non-^13^C-labeled control experiments, the δ^13^C of CO increased on average by 1.3 ± 0.2‰ between day 2 and day 60, and there was no decrease in relative CO concentrations over this period of time.

At the end of the CO utilization experiments, all bottles were frozen for PLFA identification, quantification, and stable isotope analysis (Table 2). Of the three live non-labeled bottles, one broke during freezing, so only two live non-labeled bottles were available for analysis. PLFA extractions yields were between 8.0 and 25.4 nmol PLFA per bottle, with slightly higher yields in live experiments compared to the killed controls (18.6 ± 4.8 and 12.3 ± 3.8 nmol; *t* = −2.03, *P* = 0.096 [Welch's *T*-test]) (Table 2). PLFA composition was dominated by 18:1ω 7, which made up 26.2–34.1 mol% of all PLFA. 6.9–15.0 mol% of the PLFA composition was made up of 16:0, 16:1ω 7, cy17:0, 18:0, 18:1ω 9.While cy19:0 as well as smaller amounts (3.3–5.4 mol%) of 20:0 and 22:0 made up the residual PLFA composition. There was no difference in PLFA composition between live experiments and the killed controls except for a higher abundance of 18:1w7 and a lower abundance of 18:1ω 9 in the live experiments (Table 2).

**Table 2:** **Average PLFA total concentrations, mol%, and δ^13^C values from ^13^C-labeled and non-labeled CO utilization experiments from triplicate experiments except where noted**.

	**Live**				**Killed**		**Welch *T*-test** ^**^
	Natural abundance CO^*^		**^13^C labeled CO**		**Natural abundance CO**		
	**Mean**	***SD***	**Mean**	***SD***	**Mean**	***SD***	**(*p*-value)**
**Sum of PLFA (nmol bottle^−1^)**	15.3	2.8	20.8	5.0	12.3	3.8	**0.096**
**PLFA COMPOSITION (mol%)**							
16:0	10.7%	3.1%	15.0%	2.0%	16.3%	2.7%	0.211
16:1ω7	6.9%	2.3%	13.7%	3.2%	9.2%	3.6%	0.575
cy17:0	7.1%	0.6%	7.8%	1.4%	7.8%	0.5%	0.647
18:0	11.9%	2.6%	10.3%	1.9%	10.9%	0.8%	0.953
18:1ω9	9.2%	0.7%	8.3%	1.2%	12.1%	1.8%	**0.062**
18:1ω7	34.1%	0.3%	30.0%	2.2%	26.2%	3.2%	**0.079**
cy19:0	9.7%	0.5%	7.9%	1.1%	10.4%	1.5%	0.161
20:0	5.4%	1.3%	3.7%	0.7%	3.6%	0.3%	0.235
22:0	5.1%	1.5%	3.3%	0.8%	3.5%	0.4%	0.412
**PLFA δ^13^C**							
16:0	−28.3‰	0.1‰	−25.2‰	2.7‰	−27.8‰	1.3‰	
16:1ω 7	−36.0‰	2.7‰	−22.4‰	9.4‰	−29.2‰	7.6‰	
cy17:0	−26.7‰	0.6‰	−25.3‰	1.1‰	−24.6‰	1.1‰	
18:1ω 9	−25.8‰	0.8‰	−25.0‰	0.5‰	−25.8‰	0.4‰	
18:1ω 7	−24.2‰	0.1‰	−24.8‰	0.9‰	−25.2‰	0.4‰	
cy19:0	−27.0‰	0.2‰	−26.9‰	1.0‰	−27.2‰	0.6‰	

* *The average of two live non ^13^C-labeled treatments, L2 = 13.3 nmole and L3 = 17.3 nmole. The L1 bottle broke during freezing*.

** *Welch T-tests compared live (n = 5) vs. killed (n = 3) experiments*.

*Bold p-values are values less than 0.1*.

The PLFA in killed controls likely originate from the microbial community present at the beginning of the experiment, either as part of the *in situ* community in the carbonate sediment, or grown during pre-incubation. Noteworthy, PLFA persisted in the killed controls over a prolonged period (60 days) even though pH was lowered to ~1. In contrast, the increase of biomass due to microbial growth during CO addition the experiments was either absent or very limited and not associated with a major change in PLFA composition.

The detected PLFA were either non-specific (e.g., 16:0, 18:0, 18:1ω 9, 20:0, 22:0) or typically associated with Gram negative bacteria (16:1ω 7, 18:1ω 7, cy17:0, cy19:0) supporting the dominance of Hydrogenophaga-related organisms. Noteworthy, terminally branched PLFA (e.g., iso and ante-iso 15:0) characteristic for Gram positive bacteria were not detected, even though metagenomic studies of the spring found high abundances of Erysipeloceae, a family within the Gram positive phylum Firmicutes.

**Figure 6:**
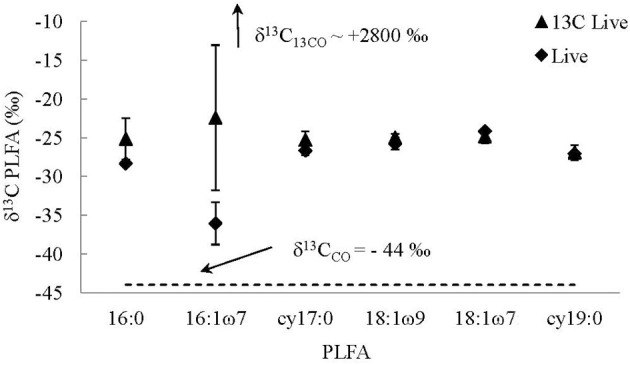
**Average δ^13^C of PLFAs from live ^13^C-labeled CO (13C Live) and live non-labeled CO (Live) treatments**. The values reported for the live ^13^C-labeled treatments are average values of replicate experiments, while the values reported for the live treatments are an average of two bottles, as one broke during freezing. The error bars are the standard deviations of the averages.

For the most part the δ^13^C of the PLFAs were similar in the live ^13^C-labeled CO compared to the live non-labeled CO experiments [i.e., incubated with CO with a natural abundance of ^13^C/^12^C (δ^13^C_CO_ = −44.4‰)] with the majority of individual PLFA (i.e., cy17:0, 18:1ω 7, 18:1ω 9, and cy19:0) having well constrained δ^13^C values between −27.2 and −24.2‰ in all treatments (Table 2, Figure 6). However, the uptake of the ^13^C into two individual PLFAs was detected, indicating that some microbial taxa incorporated CO into their biomass while others did not (Table 2, Figure 6). The PLFA associated with gram negative bacteria, 16:1ω 7, had more negative δ^13^C than other PLFA in live non ^13^C CO-labeled experiments (−38.0 ± 3.5‰ and −34.1 ± 0.4‰), likely due to the very negative δ^13^C of the CO used for the experiment (−44.4‰), and less negative but variable δ^13^C values in live microcosms incubated with ^13^C-labeled CO (−29.5 ± 0.5‰, −26.0 ± 0.5‰, and −11.8 ± 0.5‰). The same trend was present in the non-specific PLFA, 16:0, but to a much lesser extent (Table 2, Figure 6). Additionally, the δ^13^C of 16:0 and 16:1ω 7 were highly correlated (*R* = 0.94, *p* = 0.0005). However, the difference in δ^13^C between labeled and non-labeled experiments (up to 26‰) was very low compared to the high concentration in ^13^C in CO in the labeled microcosms which was greater than 2800‰, indicating that carbon that resulted from CO fixation made up <1% of PLFA.

## Discussion

### Lack of evidence for microbial methanogenesis at sites of present-day continental serpentinization

The results from the bicarbonate and organic acid addition experiments showed no microbial production of methane via autotrophic or organic acid fermentive pathways. Therefore, if methanogens utilizing these substrates are present in the spring fluids they are either not active, not active in very specific environments that were created in the lab, or that the methane production is very slow. These results support a previous geochemical study of CH_4_ and higher molecular weight hydrocarbons at the Tablelands' WHC2 pool (Szponar et al., 2013). Szponar et al. (2013) concluded that the primary source of dissolved methane in the WHC2 pool was not microbial, but most likely thermogenic or abiogenic in origin. This conclusion was based on carbon isotope values and gaseous compositions more similar to those previously found for thermogenic and abiogenic gases. This observation was consistent with initial microbiological studies of the Tablelands spring fluids, which were unable to detect archaeal 16S ribosomal RNA genes in Tablelands fluids via the polymerase chain reaction (Brazelton et al., 2013). Additionally, metagenomic sequences predicted to represent methanogens comprised only 0.2% of all metagenomic sequences from WHC2B in 2010 (Brazelton et al., 2012). Therefore, if methanogens were present in the WHC2 pool they were extremely rare. Less common microbial methane substrates, methanol, methylamines, and dimethyl sulfide, were not tested in these experiments; however, their potential contributions to microbial methane should be tested.

### Microbial CO utilization at sites of present-day continental serpentinization

In addition to carbonate, CO may also be used as a carbon substrate for autotrophic metabolisms in high pH environments. The results from the CO utilization experiments demonstrated that CO was utilized by microorganisms from the WHC2 pool. Unlike CO_2_, whose speciation is pH dependant, CO does not readily participate in hydrolysis reactions (i.e., reactions with H^+^) and therefore CO concentration/speciation is not dependent on pH. The CO oxidizing enzyme, carbon monoxide dehydrogenase (CODH), was detected in WHC2 pool fluids (Brazelton et al., 2012). This enzyme is used by organisms that utilize CO as their electron donor and their carbon source.

**Figure 7:**
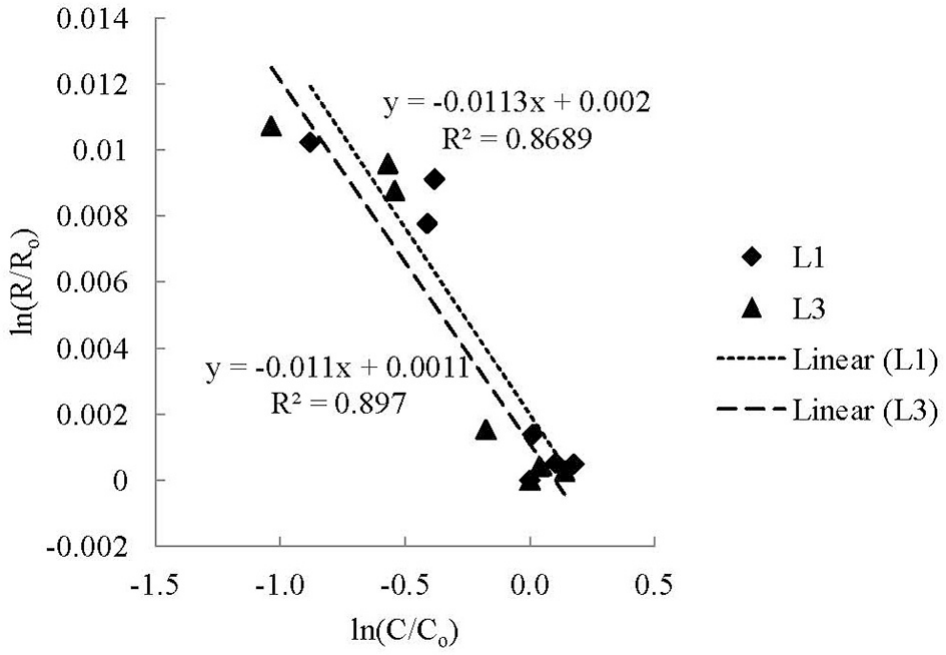
**The kinetic isotope effect (KIE) of microbial CO utilization using a Rayleigh Distillation Equation (RDE) (Mariotti et al., 1981)**. The fractionation factor (α) is equal to 1 plus the slope of a linear regression of ln(R/Ro) vs. ln(C/Co) determined from experimental data, where *R*_o_ is the initial isotopic ratio (^13^C/^12^C) of the CO and *R* is the isotopic ratio of the CO at time *t*, and C_o_ is the initial concentration of CO and C is the concentration of CO at time *t* (Scott et al., 2004). Fractionation factors are often reported as enrichment values [ε = 1000 × (α − 1)].

The results from the CO utilization experiments demonstrated that CO was utilized by microorganisms from the WHC2 pool. In the non-labeled CO experiments, the residual CO concentration decreased on average 62% over 60 days in live experiments. At the same time, the residual CO became on average 10.1 ± 1.5‰ more enriched in ^13^C in the live non-labeled CO experiments. Therefore, there was most likely a biological process occurring whereby CO containing ^12^C was utilized faster than CO containing ^13^C causing the ^13^C/^12^C ratio of the residual CO to increase over time. The kinetic isotopic fractionation can be determined using the Rayleigh Distillation Equation (RDE) (Figure 7). The isotopic enrichment factor determined for both live non-labeled CO addition experiments were fairly consistent (−11.3 and −11.0‰) (Figure 7), demonstrating that the isotopic fractionations in both experiments were consistent. The correlation coefficients for the regression lines were 0.8792 and 0.8970, indicating that the data were well described by the RDE in both experiments; however, the high *R*^2^ values are driven by binomial dispersion of the data (Figure 7). Additional experiments should be performed to continue to quantify the kinetic isotope fractionation of the microbial CO utilization in these systems. The RDE applied to the killed controls had very poor correlation coefficients for the regression lines (averaging 0.1608). This is not surprising as the δ^13^C of CO remained fairly constant in the killed controls, while the concentrations varied.

This is the first study to report CO consumption and its isotopic enrichment factors in ultra-basic springs associated with present-day serpentinization. There are few measurements of the isotopic enrichment factors of biological uptake of CO to compare this value to; however, the values estimated in this study are slightly larger compared to other microbial CO utilization studies. For example, the enrichment factors determined during CO consumption in soil was determined to be −8‰ (Tsunogai et al., 2002), while the isotopic enrichment factor during marine microbial oxidation of CO was −5‰ (Nakagawa et al., 2004).

The PLFA data from the ^13^C-labeled CO utilization experiments indicated some microbial uptake of CO (Table 2). The total amount of PLFA (nmol/bottle) were slightly higher in the live experiments compared to the killed controls. The PLFA identified were the same in both the live experiments and killed controls, and there was no difference in PLFA composition between live experiments and the killed controls except for a higher abundance of 18:1ω 7 and a lower abundance of 18:1ω 9 in the live experiments, indicating that there was little to no community change during the CO addition experiments. The18:1ω 7 PLFA is typically associated with Gram negative bacteria, while the 18:1ω 9 occurs widespread in bacteria and fungi (Zelles et al., 1992; Ruess and Chamberlain, 2010). However, ^13^C isotopic enrichment in the live ^13^C-labeled CO utilization experiments was not observed in the 18:1ω 7 PLFA, but instead the 16:1ω 7 which is also associated with Gram negative bacteria. These results support limited incorporation of carbon from CO likely by Gram negative bacteria. Based on the assumption that 1% of the entire microbial biomass in the experiments was derived from CO carbon, less than 2% of the total CO carbon consumed in the experiments (64.7 μg) was incorporated into the microbial biomass (based on 363.6 nmol PLFA per mg biomass-C, Frostegård and Baath, 1996). This indicates that in our experiments, CO was used primarily as a source of energy, not biomass carbon. This might have resulted from the limited biomass increase during the experiment and/or because CO oxidizers utilized other carbon sources present in the ultra-basic fluid such as acetate and formate that were detected in the spring fluids (Table 1). However, PLFA carbon might have over-proportionally originated from acetate because acetyl-CoA is the universal precursor for fatty acid biosynthesis and therefore the incorporation of CO derived carbon into PLFA might underestimate the incorporation into overall biomass. Also, since microbial growth during the experiment was limited, turn-over of PLFA might under represent the turn-over of the bulk biomass. Nevertheless, even if this led to an underestimation of the carbon incorporation into biomass by an order of magnitude, CO-derived carbon would still account for only a small fraction of the total biomass or the total CO loss during the experiment.

The utilization of acetate and formate is supported by the field data. Acetate and formate concentrations were lower in the pool water compared to the recharging groundwater, while dissolved H_2_ concentration stayed the same in the pool and recharge waters (Table 1). This suggests that the acetate and formate were preferentially consumed in the pool, while H_2_ was not consumed in the pool. CO concentrations were not detected; however, this is not unexpected, because our CO detection limit (~10 μM) is orders of magnitude greater than the upper range of CO concentrations (0.2–20 nmoles/L) measured in groundwaters across the US (Chapelle and Bradley, 2007).

The Comamonadaceae which dominate the sediments and overlying fluids of the WHC2 pool (Figure 2) are Gram-negative bacteria, and they have been found at many sites of serpentinization and other high pH environments (Schrenk et al., 2013; Suzuki et al., 2014). Their closest characterized relatives include the *Hydrogenophaga*, which are aerobic or facultatively anaerobic. At least some *Hydrogenophaga* species can grow autotrophically on H_2_ or CO if organic carbon is not available (Willems et al., 1989). However, in the case of an environment where organic acids are present, like the Tablelands, it is possible that CO may be used as an electron donor by *Hydrogenophaga*-like bacteria but not as a carbon source for autotrophic growth. Unfortunately, H_2_ and organic acid concentrations were not monitored during our experiments; however, further experiments that control and monitor organic acids, CO, and H_2_ concentrations should be performed.

## Conclusions

Fluids discharging from sites of present-day continental serpentinization are ultra-basic, reducing, and low in electron acceptors, creating a challenging environment for life as we know it. However, once these fluids are discharged at the surface additional inputs such as oxygen and nutrients can enter the system, creating high redox gradient environments, which may no longer be nutrient limited. While the results from this study did not find evidence for microbial methanogenesis with organic acid and bicarbonate substrates, it did find empirical evidence in support of microbial CO utilization in agreement with earlier metagenomic studies. The CO utilization may be occurring when ultra-basic reducing waters associated with serpentinization mix with oxygenated surface waters. Microbial CO utilization may also occur at other sites where groundwater associated with serpentinization comes in contact with the atmosphere or mixes with oxygenated waters. For example, CO utilization may be possible at other serpentinization sites such as Cabeco de Vide, Portugal (Tiago and Verissimo, 2013), and The Cedars (Suzuki et al., 2014) where *Hydrogenophaga*-like organisms have been found.

### Conflict of interest statement

The authors declare that the research was conducted in the absence of any commercial or financial relationships that could be construed as a potential conflict of interest.
